# Order and interactions in DNA arrays: Multiscale molecular dynamics simulation

**DOI:** 10.1038/s41598-017-05109-2

**Published:** 2017-07-06

**Authors:** Julija Zavadlav, Rudolf Podgornik, Matej Praprotnik

**Affiliations:** 1 Department of Molecular Modeling, National Institute of Chemistry, Hajdrihova 19, SI-1001 Ljubljana, Slovenia; 2 Department of Physics, Faculty of Mathematics and Physics, University of Ljubljana, Jadranska 19, SI-1000 Ljubljana, Slovenia; 3 Theoretical Physics Department, J. Stefan Institute, Jamova c. 39, SI-1000 Ljubljana, Slovenia; 4 Chair of Computational Science, ETH Zurich, Clausiusstrasse 33, CH-8092 Zurich, Switzerland

## Abstract

While densely packed DNA arrays are known to exhibit hexagonal and orthorhombic local packings, the detailed mechanism governing the associated phase transition remains rather elusive. Furthermore, at high densities the atomistic resolution is paramount to properly account for fine details, encompassing the DNA molecular order, the contingent ordering of counterions and the induced molecular ordering of the bathing solvent, bringing together electrostatic, steric, thermal and direct hydrogen-bonding interactions, resulting in the observed osmotic equation of state. We perform a multiscale simulation of dense DNA arrays by enclosing a set of 16 atomistically resolved DNA molecules within a semi-permeable membrane, allowing the passage of water and salt ions, and thus mimicking the behavior of DNA arrays subjected to external osmotic stress in a bathing solution of monovalent salt and multivalent counterions. By varying the DNA density, local packing symmetry, and counterion type, we obtain osmotic equation of state together with the hexagonal-orthorhombic phase transition, and full structural characterization of the DNA subphase in terms of its positional and angular orientational fluctuations, counterion distributions, and the solvent local dielectric response profile with its order parameters that allow us to identify the hydration force as the primary interaction mechanism at high DNA densities.

## Introduction

Behavior of (double-stranded) dsDNA in the *biological milieu* is seldom mimicked by its properties in dilute solutions^1, 2^. At elevated densities, ubiquitous in eukaryotic nuclei, bacterial nucleoids and/or viral capsids, the properties of dsDNA are in a crucial way connected with the detailed interactions between the double stranded helices, exhibiting increasingly detailed features as the average separation between DNAs decreases and closely apposed molecules sense progressively finer molecular details^3^. In parallel with the molecular identity of the DNA itself, the nature of the bathing solution medium makes its indelible mark not only on the quantitative details, but also on the *qualitative type* of the interactions between DNA molecular surfaces, as probed by the osmotic stress method^4^ (see Supplementary Information (SI)). Varying the DNA concentration results in a rich phenomenology, usually quantified by the equation of state (EoS), connecting the DNA concentration, as measured e.g. by SAXS, and the DNA osmotic pressure. For monovalent salt solutions the EoS exhibits a monotonic behavior, characterized by the hydration and fluctuation enhanced electrostatic regimes punctuated by a small discontinuous DNA density change between the cholesteric and hexatic phases and between the hexatic and orthorhombic phases for long fragment DNA^4^, the exact sequence and identity of the phases depending on the DNA length^5, 6^. For multivalent counterions at concentrations below a critical value depending on their identity^7^, the EoS exhibits more pronounced van der Waals-like density discontinuities, signaling a buildup of *counterion*-*mediated attractive interactions*, whose details can be inferred from complementary experiments^8^ (see SI), that eventually lead to DNA condensation above the critical multivalent counterion concentration^9^.

The phenomenology of DNA molecular interactions themselves, even if obviously complicated, does not exhaust the description of high density DNA solutions. Details of the solution exposed molecular surface, distribution of charges and available water hydration sites, consistent with the characteristic molecular symmetries, induce ordering not only in the average position of the DNA centroids, but also ordering of their relative orientations, ordering of the vicinal solvent molecules and ordering of the strongly interacting counterions that exhibit sudden discontinuous changes in the associated order parameters, signaling *phase transitions* characterized by different types of molecular order^10, 11^. On increase of DNA density a progressive *sequence of mesophase ordering* has been identified that consists of: isotropic (i) → cholesteric (ch) → line hexatic (lhex) or hexagonal columnar (hex) → orthorhombic (orto) phase^3, 5, 12–15^, with many details, including the complete DNA length dependence and the demarcation between the line hexatic and hexagonal order, still remaining to be systematically investigated^16^. The ultimate justification for the study of these phases is their particular relevance for DNA compactification which enables a *μ*m to cm long molecule to fit into a cell nucleus, a bacterial nucleoid or a virus capsid.

Direct experiments probing the interactions and/or the associated molecular order can not provide all the desirable features that would allow an unequivocal deconvolution of the main physical mechanisms underlying them, and theoretical interpolations connecting one with the other are therefore necessary^17^. While theories of DNA interactions abound (see SI), based mostly on an increasingly more sophisticated description of the electrostatic interactions^18^, spanning the regime of simple electrolyte screening and then all the way to the counterion correlation-driven attractions in a multivalent ion-DNA system^19^, the nature of the interaction-induced ordering was much less prone to theoretical scrutiny^20^. The continuum modeling^19–22^ and coarse grained simulation approaches^23–31^, which mostly underpin these theoretical endeavors, certainly led to important insights, especially in elucidating the significance of the counterion valency for the emergence of correlations, reversing the sign of the electrostatic interactions between charged helices, that in its turn leads to DNA condensation and precipitation. However, the difficulties connected with the full implementation of molecular details of the DNA solution exposed surface, the granularity of the molecular solvent, and the interactions between both and the mobile charges in solution, together with the extraordinary demands they make on available computer resources, hamper the ambitions to understand all the relevant molecular details”.

As a consequence, detailed all-atom molecular dynamics (MD) simulations appear to be the only vehicle that can bring fourth a *proper understanding* of the mechanisms and the relevant couplings between them^32, 33^, leading to the experimentally observed interaction and ordering phenomenology of DNA at intermediate and high density mesophases. On the downside, all-atom MD simulations of such complicated systems require huge computational resources and majority of simulations aiming to describe details of the DNA solution phenomenology consist of 1–3 DNA molecules, where the focus is the counterion binding, effective interactions between DNA molecules, and/or azimuthal dependence of DNA-DNA interaction^26, 34–40^. Only recently it became feasible to set up a realistic all-atom MD simulation, with properly parameterized and tested molecular potentials that could be applied to a larger set of DNA molecules^41–43^, describing a condensed DNA array in the presence of counterions and salt, characterized by a single packing geometry, but as yet with only partial characterization of the DNA countercharge and solvent ordering. In this respect, the full characterization of concentrated DNA solutions at different densities, including the mono- and multivalent counterions, salt and explicit molecular solvent together with ordering transitions between the density dependent mesophases at atomic resolution is still to a large extent missing.

Generalizing the previous simulation efforts, we analyze two types of DNA arrays with hex and orto local packing symmetries in 1 M NaCl salt, corresponding to inverse Debye screening length *κ* = 3.25 nm^−1^. The system is charge neutralized with either pure Na^+^ counterions or a combination of Na^+^ and the naturally occurring condensing agent spermidine Spd^3+^, where 6 Spd^3+^ molecules are used per each DNA molecule of 3.4 nm length in the simulation cell. We not only analyze the pertaining interaction phenomenology in a finite orientationally ordered array with different local packing symmetries, but also characterize the positional and angular orientational order of the DNA sub-phase as well as employ the Lindemann criterion^44^ to extract the osmotic pressure of the *phase transitions* between phases of different order and symmetry, all concurrently with the distribution of counterions and positional, orientational and local tetrahedral coordination order of the water molecules. Our aim here is thus to provide an *exhaustive tableau* of the couplings between the interactions and order in the high density DNA mesophases and phase transitions between them.

**Figure 1:**
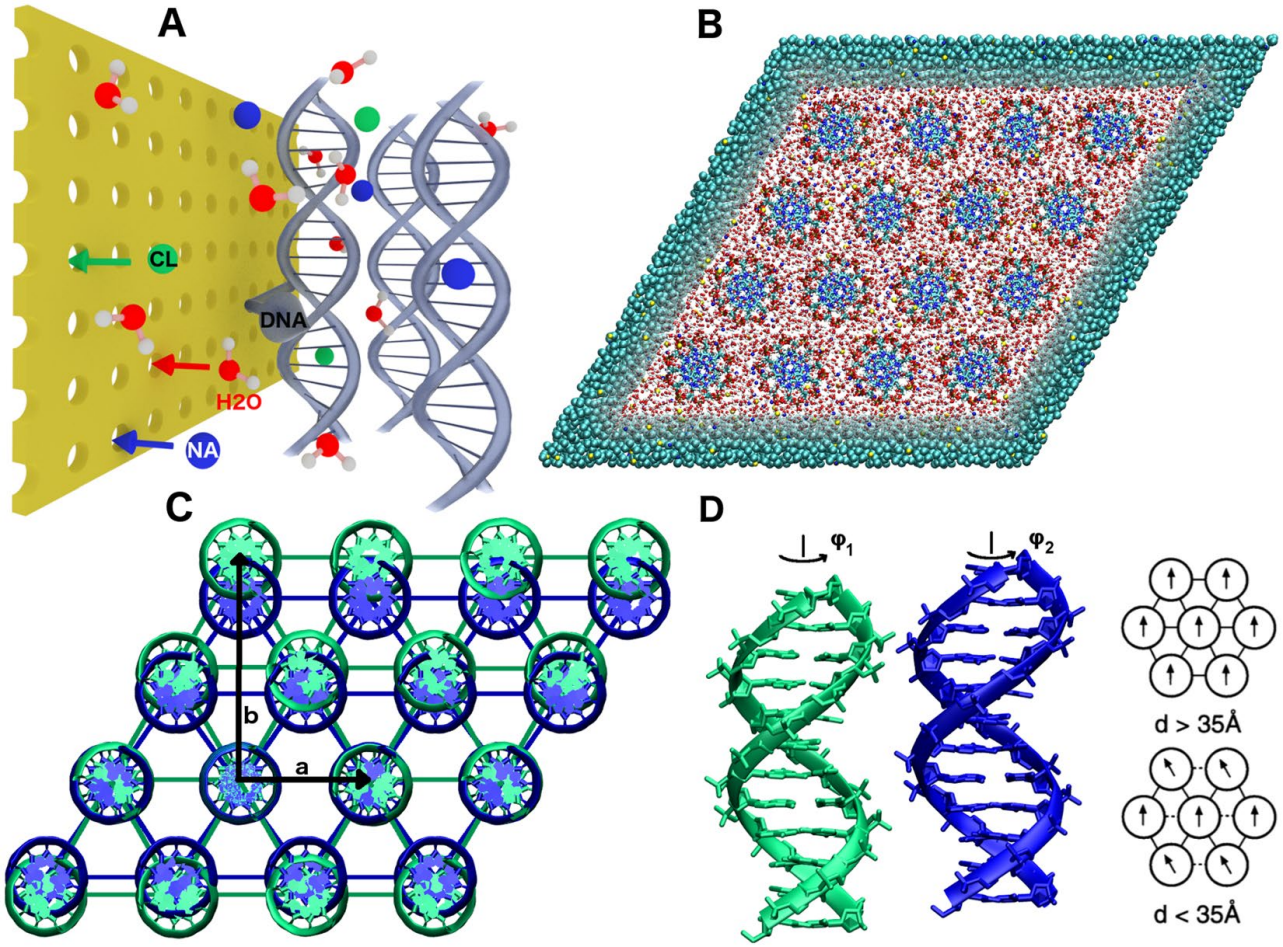
(**A**) Sketch of the simulation setup. An array of DNA molecules is enclosed within the semipermeable membrane, which is permeable to solution molecules (i.e., water, Na^+^, and Cl^−^), but impermeable to the DNA molecules. (**B**) Top-down view of the simulation box containing 16 DNA molecules 3.4 nm long on a hexagonal lattice, to demonstrate the multiscale simulation setup, where the solvent molecules are modeled at two levels of resolution, i.e., the atomistic resolution in the rhombic region containing the DNA molecules and the coarse-grained representation outside. The water molecules and ions (Na^+^ and Cl^−^) can change their resolution adaptively on the fly between the atomistic and coarse-grained regions, whereas the DNA array is at all times modeled with the atomistic resolution. To match the experimental setups, the DNA molecules are periodic in the longitudinal direction and thus effectively infinite. (**C**) Superimposed (on DNA molecules in the second row) hex and orto lattices defined by lattice parameters *a* and *b*. The ratio *b*/*a* of orto phase was set to 1.43. Note, that the angle formed by the DNA array is 60° and 55° for the hex and orto lattices, respectively. The semipermeable membrane enclosing the array is tilted at the same angle as the array. (**D**) Modified from ref. 93: Illustration of an optimal orientation between two DNA molecules, whose orientations are characterized by angles ${\varphi }_{1}$ and ${\varphi }_{2}$. In a densely hexagonally packed DNA array, corresponding to small interaxial spacings *d* between DNA molecules, the nonzero optimal orientational angle between the molecules leads to angular frustrations.

Since our focus is primarily on the structural characterization of the molecular order in the hex and orto phases and on the driving mechanism(s) of the phase transition between them, we perform extensive MD simulations of DNA arrays with the two local packing symmetries (see Fig. 1). To obtain the EoS we perform multiple simulations with varied DNA-DNA densities for each bathing solution composition and in contrast to previous work^42^ focus exclusively on high DNA densities as these are relevant for the hex-orto phase transition. To conduct the simulations (overall several hundred ns long) efficiently, we resort to a multiscale MD technique *AdResS* (*Adaptive Resolution Scheme*)^45, 46^, which has been already successfully applied to various biological systems^47–52^. This methodology enables a concurrent and consistent coupling between the atomistic and the coarse-grained representations with a key feature of allowing molecules to freely move not only in real space but also in the *resolution space* across different regions and change their resolution on the fly according to their position in the computational domain.

## Methods

### AdResS

The adaptive resolution scheme (AdResS)^45, 46^ is an MD simulation technique that enables a concurrent coupling between two domains where MD simulations can be performed by different force fields, e.g., atomistic and coarse-grained. When a coarse-grained molecule leaves the coarse-grained domain it is remapped into the atomistically resolved molecule with a random orientation. To avoid any overlaps of its atoms with the atoms of the neighboring molecules, it is required that the introduction of the atomistic degrees of freedom be continuous and not instantaneous. To this end, an interface layer between the atomistic and coarse-grained regions is introduced that allows an atomistic molecule to gradually find an energetically permissible orientation with respect to its neighboring molecules. The interface region, also called a hybrid region, contains hybrid molecules where both representations are superimposed. The coupling between different levels of resolution is achieved via a force interpolation scheme. The total force acting on a molecule *α* is given by

1 $$\begin{array}{rcl}{{\bf{F}}}_{\alpha } & = & \sum _{\beta \ne \alpha }\,w(|{{\bf{R}}}_{\alpha }-{\bf{R}}|)w(|{{\bf{R}}}_{\beta }-{\bf{R}}|){{\bf{F}}}_{\alpha \beta }^{ex}\\  &  & +\sum _{\beta \ne \alpha }\,[1-w(|{{\bf{R}}}_{\alpha }-{\bf{R}}|)w(|{{\bf{R}}}_{\beta }-{\bf{R}}|)]{{\bf{F}}}_{\alpha \beta }^{cg}\\  &  & -{{\bf{F}}}_{\alpha }^{TD}(|{{\bf{R}}}_{\alpha }-{\bf{R}}|),\end{array}$$

where ${{\bf{F}}}_{\alpha \beta }^{ex}$ and ${{\bf{F}}}_{\alpha \beta }^{cg}$ are the forces between molecules *α* and *β*, obtained from the atomistic and coarse-grained potentials, respectively. The sigmoidal function $w\in [0,1]$ is used to smoothly couple the high and low resolution regimes, where **R**_*α*_ and **R**_*β*_ are two-dimensional (x, y) vectors of centers of mass (COMs) of molecules *α* and *β*, respectively. The **R** is a two-dimensional (x, y) vector to the nearest point on the atomistic-hybrid boundary. Here, we employ the rhombic boundaries between the resolution region boundaries. Since the atomistic-hybrid boundary is placed behind the semi-permeable membrane the DNA molecules are at all times modeled at the atomistic scale. Note, that the edges of the hybrid rhombus are rounded. The thermodynamic (TD) force ${{\bf{F}}}_{\alpha }^{TD}$ is needed for the compensation of the difference in the chemical potential of atomistic and coarse-grained resolutions^53, 54^. For each molecule type, the TD force is computed with an iterative procedure as described in detail in refs 48, 53–55 and applied to molecules in the hybrid region (see SI for further details). To supply or remove the latent heat due to the switch of resolution, the method requires the use of a local thermostat^45^.

Many recent AdResS advances are addressing the computation of statistical mechanical quantities, e.g., entropy, free energy, chemical potential, which are needed for studying phase transitions^56–60^. The Hamiltonian formulation of AdResS^61^ permits also the adaptive resolution Monte Carlo simulations^62^. Current efforts are also focusing on combining AdResS and advance sampling techniques, e.g., metadynamics^63^. Another direction of AdResS development is considering simulations of open grand-canonical systems^64^, e.g., the grand-canonical (GC)-AdResS^65, 66^ and open boundary molecular dynamics (OBMD) approaches^67, 68^. To properly model the hexagonal to orthorhombic phase transition all of these techniques would need to be combined. However, the current stage of this method development is still premature for studies of this kind of complex systems. Therefore, as already mentioned, in this study, to circumvent the problem of exhaustive free energy computation, we resort to osmotic pressure computation and the Lindemann criterion to determine the point of phase transition. Since the DNA arrays are enclosed within the semi-permeable membrane, which allows the passage of water and salt ions, the system setup is mimicking an OBMD-like system.

### Simulation setup and computational details

Experimental results^4, 5^ point to a DNA liquid crystalline phase transition from the ch → hex lattice at an approximate DNA concentration 380 mg ml^−1^, which corresponds to interaxial lattice spacing of 3.15 nm. On the other hand, the hex → orto phase transition is observed at DNA-DNA interaxial spacings equal to 2.37 nm (670 mg ml^−1^). Additionally, the distortion from the hex lattice (ratio of lattice parameters $b/a=\sqrt{3}$; see Fig. 1) increases with DNA concentration, with the highest observed distortion equal to the ratio of 1.43. Based on these findings, we explore the hex and orto lattices of DNA assemblies at DNA-DNA interaxial spacings that range from 2.0 (2.1 for the orto lattice) to 3.6 nm. To observe the utmost difference between the two lattices, we set the ratio *b*/*a* of orto phase to 1.43. The DNA molecules are immersed in 1 M NaCl bathing salt solution, while additional Na^+^ counterions or a combination of Spd^3+^ and Na^+^ counterions are added to neutralize the negative phosphate charges of the DNAs. We simulate 16 DNA molecules (for a discussion on the sufficient number of DNA molecules see SI), one helical pitch long, i.e., of length 3.4 nm. However, since the periodic boundary conditions are used also in the longitudinal direction and the 5′ and 3′ ends of the chains are linked, we are effectively simulating infinitely long DNA molecules^48^, the simulation setup thus corresponding more closely to the experiments on oriented long DNA fibers. The imposed DNA periodicity fixes the helical twist of DNA and prevents any major bending fluctuations. Consequently, the DNA’s persistence length, which is about 50 nm and much longer than one DNA pitch, is effectively altered. However, previous simulations with periodic DNA fragments have shown stable, B-form DNA structures that still have a lot of freedom for variation of the local structure^35, 69^. Moreover, to model the DNA on a persistence length scale one has to resort to coarse-grained models like in ref. 70. By the very nature of our simulation setup, we cannot expect to capture the full effect of the bending fluctuations, known to contribute to the equation of state at smaller densities, but not at the densities we investigate here^4, 17, 71^. The finite size effects in plane orthogonal to the DNA helix due to the boundary conditions are studied by evaluating the osmotic pressure as a function of the system size, i.e., the number of DNA molecules. As shown in Fig. 3 of SI, the osmotic pressure results for different sizes are within the standard deviation. Furthermore, we would like to stress that for the DNA molecules the periodicity is only imposed in the direction along the long axis of DNA, i.e., the solution buffer around the DNA array is large enough so that DNA molecules do not see each other across the simulation box.

The whole DNA array is at all times modeled at full atomistic resolution, but since the region outside the semipermeable walls is used only as a solution reservoir, it can be modeled with the coarse-grained resolution, acting in a similar way as a buffer in an OBMD simulation^67, 68^. We perform our multiscale simulations, with a length of 22 ns (2 ns equilibration followed by 20 ns production runs) for each system or 900 ns of production runs in total, employing AdResS. The latter is coded in the ESPResSo++ software package^72^ and the simulations are run on the local linux cluster^73^. Newton’s equations of motion are integrated by the standard Velocity-Verlet integrator with a 1 fs timestep. The length of the production runs is 20 ns for each considered system or 900 ns in total. Water geometry is constrained with the SETTLE algorithm^74^, while the hydrogen atoms of DNA molecules are constrained with RATTLE^75^. The temperature is kept constant at 300 K with the use of the Langevin thermostat with a coupling constant 5.0 ps^−1^. The non-bonded interactions are calculated within a cutoff distance of 0.9 nm. The generalized reaction field method^76^ is used for the electrostatic interaction beyond the cutoff, with dielectric permittivity of outer region equal to 80 and the inverse Debye screening length *κ* = 3.25/nm corresponding to a 1 M salt solution. The dielectric permittivity of the inner region, that is, within cutoff distance, is equal to 1 and 80 for the atomistic and coarse-grained regions, respectively. This ensures that the ion-ion interactions are properly screened in the coarse-grained region, where we use the same electric charges for the salt ions as in the atomistic region, i.e., the ions thus interact via the same potentials in the atomistic and coarse-grained models aside from the changed dielectric permittivity. We compute the osmotic pressure by introducing a semi-permeable membrane around the set of DNA molecules and apply interaction to the DNA backbone atoms. This prevents a given DNA molecule from crossing the wall, whereas the solvent molecules can freely pass through^77, 78^ (see SI for further details).

## Results and Discussion

### Osmotic pressure

**Figure 2:**
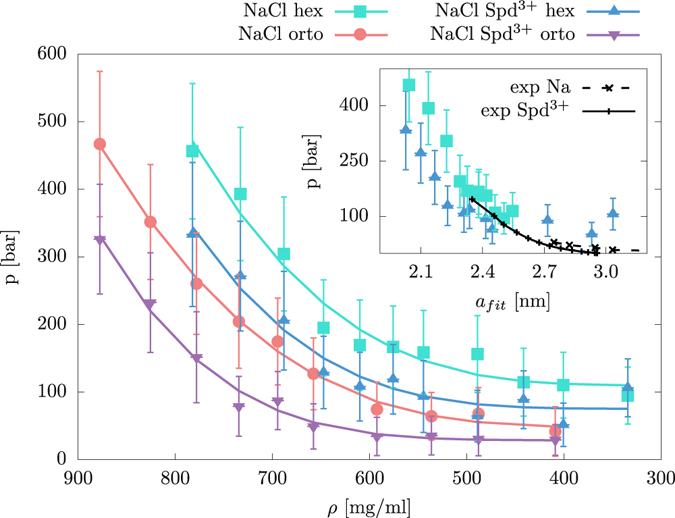
Osmotic pressure (mean and standard deviation) of DNA arrays as a function of the DNA density. The results are shown for hex and orto lattices and at two bathing solution setups (1 M NaCl concentration with pure Na^+^ counterions and mixed Na^+^/Spd^3+^ counterions). The exact results are shown with points, while the lines are used to guide the eye and show the exponential fit. Every point corresponds to a separate simulation. Inset: Osmotic pressure (mean and standard deviation) of the hexagonal array with Spd^3+^ counterions as a function of fitted (to mean DNA positions) lattice parameter *a*. The black dotted and full lines correspond to the experimental observations in the presence of monovalent Na and multivalent Spd^3+^ solution, respectively^8, 89^.

The osmotic pressure of a DNA array is evaluated by surrounding it with a semipermeable membrane and measuring the force exerted on this membrane. Figure 2 shows the results for the four investigated cases (two packing symmetries and two bathing solution conditions). Since Spd^3+^ in all investigated cases is at subcritical condensation concentrations (6 Spd^3+^ molecules per each DNA molecule), the osmotic pressure for all cases is monotonic, corresponding to net repulsive interactions.

**Figure 3:**
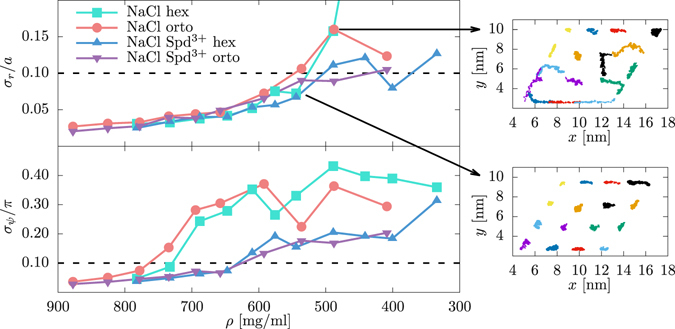
Positional (divided by lattice parameter *a*) and rotational (divided by *π*) standard deviations of mean values as a function of DNA density for the hex and orto lattice with pure NaCl and mixed NaCl and Spd^3+^ counterions. The horizontal dotted lines indicate the typical 0.1 *Lindemann threshold* value. The insets show the COM of DNA molecules in the *xy* plane, as monitored during a 20 ns simulation, for the two sizes of the hex lattice with pure NaCl counterions as indicated by the arrows.

For low DNA concentrations, the osmotic pressure does not depend on a specific type or valency of the counterions present, while at small separations the difference between two different ion types is substantial and for both lattices the presence of multivalent Spd^3+^ results in substantial lowering of osmotic pressure. The inset of Fig. 2 shows the comparison with the experimentally observed values at 2 mM Spd^3+^ ^8^ for the hex lattice. At small DNA separations the osmotic pressure values compare quantitatively with experiments, whereas at larger separations the pressure obtained with simulations is overestimated. At the lowest examined DNA concentration, the initial lattice in the simulated array is no longer preserved, as can be seen in the insets of Fig. 3. As discussed below, we find these states to be in positionally disordered phase. Hence, it is questionable whether they can be meaningfully compared to the experimental data in this density regime. The observed discrepancies between the osmotic pressure and the melting density in the simulations and in the experiment could be easily due to the small size of the simulated array as well as possibly the inaccuracies in the force field.

The observed independence of the osmotic pressure on the ion type at low DNA densities is connected with pronounced ionic screening of electrostatic interactions at 1 M salt conditions beyond the DNA intersurface separation of ~0.3 nm and is consistent with experiments^79^. In the opposite regime of high DNA densities, the osmotic pressure is quantitatively consistent with the experimental hydration force pressure^4^ and follows the concurrent variation of the water order parameter profiles (see below) just as in the case of hydrated polar surfaces in general^80^.

### Translational and rotational fluctuations

Next, we examine the details of the translational order of the DNA assemblies. To quantify the translational order we monitor the (*x*, *y*) motion of COMs of DNAs and calculate the normalized root-mean-square deviation *σ*_**r**_/*a*, where *a* is the lattice parameter (see Fig. 1C) and

2 $${\sigma }_{{\bf{r}}}=\frac{1}{{N}_{{\rm{DNA}}}}\sum _{i=1}^{{N}_{{\rm{DNA}}}}\,\sqrt{\frac{1}{{N}_{t}}\sum _{t=1}^{{N}_{t}}\,{[{{\bf{r}}}_{i}(t)-\langle {{\bf{r}}}_{i}\rangle ]}^{2}}.$$

The 〈**r**_*i*_〉 and **r**_*i*_(*t*) are the mean and the instantaneous 2D position vectors of the *i*-th DNA COM at time *t*, respectively. The obtained values as a function of DNA density are reported in Fig. 3 (see SI for *σ*_**r**_/*a* as a function of pressure). At high and intermediate concentrations, DNA molecules preserve the initial lattice and display very small translational fluctuations about their mean values, with *σ*_**r**_/*a* quite insensitive to the lattice and counterion type. As the DNA concentration decreases, the fluctuations get progressively enhanced. At the lowest concentrations, the lattice ordering is no longer preserved and positional order melts away, i.e., the systems progressively displays the properties of a positionally disordered phase. The computed ratio *σ*_**r**_/*a* can be used with an empirical 2D Lindemann melting criterion^44^, indicating a phase transition between the hex, positionally ordered, and ch, positionally disordered phases, when this ratio exceeds a certain critical value, typically taken as 0.1. We thus find that the 2D positional order melts at ≈500 mg ml^−1^. The obtained DNA density is somewhat higher than the experimentally observed value 380 mg ml^−1^ for the hex → ch transition^5^.

At small separations, the interaction potential depends crucially on the mutual (polar) orientation of the two interacting molecules around their long axes^10^. Experimentally, with increased DNA concentration, a progressive longitudinal and consequently polar orientational ordering between neighboring DNA helices in the hex phase indicates a continuous transition from a 2D hexagonal phase to a 3D hexagonal phase in the case of short fragment DNAs^5^. Several studies analyzed the orientational order of DNA mesophases^10, 14, 81^, specifically concluding that the hex packing introduces angular frustrations between neighboring DNA molecules since the optimal orientation cannot be accommodated within this local symmetry. These angular frustrations can be alleviated by a change of local symmetry towards the orto lattice, allowing two nearest neighbor molecules to maintain the optimal angle, whereas the third molecule moves further apart and can be in a non-optimal orientational configuration with low(er) energy penalty. This implies that the minimum energy states of 6 first DNA neighbors are rotated by 2*π*/3 in the case of hex packing, whereas the 4 first DNA neighbors are rotated by *π*/2 in the orto packing (see SI).

We analyze the orientational order in a similar fashion as the translational order, i.e., by computing the rotational root mean square deviations ${\sigma }_{\phi }/\pi$

3 $${\sigma }_{\phi }=\frac{1}{{N}_{{\rm{NN}}}}\sum _{j=1}^{{N}_{{\rm{NN}}}}\,\sqrt{\frac{1}{{N}_{t}}\sum _{t=1}^{{N}_{t}}\,{[{\rm{\Delta }}{\phi }_{j}(t)-\langle {\rm{\Delta }}{\phi }_{j}\rangle ]}^{2}},$$

where $\langle {\rm{\Delta }}{\phi }_{j}\rangle$ and ${\rm{\Delta }}{\phi }_{j}(t)$ are, respectively, the mean and instantaneous relative orientations of the *j*-th nearest neighbor DNA pair at time *t* computed with

4 $${\rm{\Delta }}\phi =\frac{1}{{N}_{{\rm{BP}}}}\sum _{k=1}^{{N}_{{\rm{BP}}}}\,[{\phi }_{k,1}-{\phi }_{k\mathrm{,2}}+2\pi ({z}_{k\mathrm{,1}}-{z}_{k\mathrm{,2}}){L}_{z}].$$

${\phi }_{k\mathrm{,1}}$ and ${\phi }_{k\mathrm{,2}}$ are the (polar) azimuthal angles of the *k*-th base-pair at heights *z*_*k*,1_ and *z*_*k*,2_ of the DNA molecules 1 and 2, respectively. We define the azimuthal angle as the angle between the vector pointing from the purine C8 to pyrimidine C6 carbon atoms in a base-pair and the *x*-axis of the simulation box. Since in our simulation setup the DNA molecules are effectively infinitely long, the relative orientation does not only vary with base-pair rotation but also translation along the DNA long axes, in fact, the translational and rotational motions of molecules are coupled in screw fluctuations^82^. With the last term in Eq. 4, where the *L*_*z*_ = 3.4 nm is the helical pitch (the height of simulation box is exactly one pitch), we normalize the difference in azimuthal orientation of the two DNAs to the same height level.

The ${\sigma }_{\phi }/\pi$ values as a function of DNA density are plotted in Fig. 3 (see SI for ${\sigma }_{\phi }/\pi$ as a function of pressure). We first notice different behavior pending on the mono vs. multivalent counterions. In particular, for both lattices the presence of Spd^3+^ *attenuates* the azimuthal fluctuations, an observation that can be rationalized by the *bridging* of Spd^3+^ counterions between DNA molecules as proposed already before, based on continuum models^83, 84^ as well as atomistic simulations^42^. In this case, each Spd^3+^ molecule shared by neighboring DNAs would act as an *azimuthal lock* between neighboring DNA molecules. To elucidate this conjecture, we compute the average occupancy and residence times of counterions Na^+^, Spd^3+^ and oxygen atoms of water (see SI), respectively. In systems containing only Na^+^ ions, the occupancy of oxygen atoms of water is increased, following closely the increase of the lattice parameter *a*, while the residence time decreases and varies substantially (up to 50% for the backbone). For both lattices, the occupancy and residence times at large interaxial spacings approach the value found for a single DNA molecule^48^. Similar behavior is found also for Spd^3+^ counterions. In particular, we observe that the Spd^3+^ ions bind preferentially with terminal nitrogen atoms, which suggests that the Spd^3+^ molecules are shared between DNA molecules, i.e. *bridge the space between them*, rather than absorb tightly to the surface of a single DNA molecule. In addition, it is interesting that Spd^3+^ is observed to bind more to the phosphate group than to the groove, while a reverse situation is commonly observed in simulations of a single DNA molecule.

Compared to translational order, the azimuthal orientational order is lost at much higher DNA concentrations. The ${\sigma }_{\phi }/\pi$ parameter can be used in a rotational analog of the Lindemann empirical criterion. Taking again the 0.1 threshold value we observe the loss of orientational ordering at ≈750 mg ml^−1^ and 650 mg ml^−1^ for the pure Na^+^ and Na^+^-Spd^3+^ counterion conditions, respectively, roughly at the experimentally detected hex → orto phase transition^5^.

### Phase Transition

**Figure 4:**
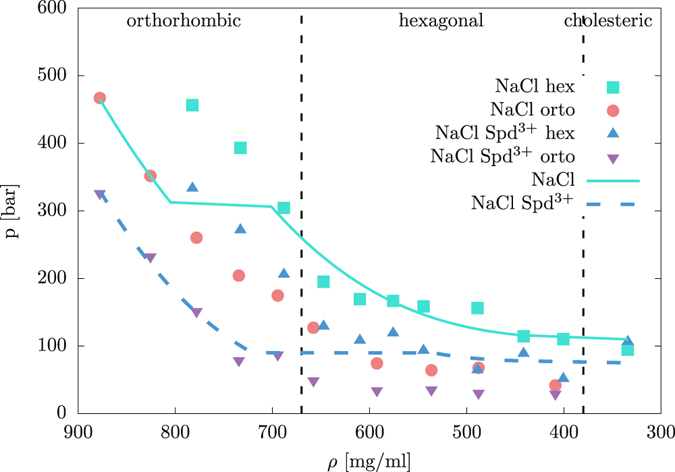
Same as Fig. 2. Lines are used to guide the eye and indicate the transition between the hex and orto phases obtained from the Lindemann criterion corresponding to angular fluctuations. For comparison, we add the experimentally observed domains of the orto, hex and ch phases^5^.

We consider rotational Lindemann criterion to be a good proxy for the hex → orto phase transition, as the rotational correlations appear to be of crucial importance in orto lattices and the loss of azimuthal ordering is found at approximately the same DNA concentrations as the phase transition. Taking the mean osmotic pressure at the Lindemann threshold (see Fig. 3 bottom and Fig. 4), the transition between the hex and orto phases then occurs roughly at DNA concentrations between 700–800 and 500–700 mg/ml for the Na^+^ and Na^+^-Spd^3+^ counterions, respectively. The obtained concentrations compare favorably with the experimental ones, i.e., ≈670 mg/ml^4, 5^. The Lindemann criterion for positional and orientational fluctuations thus works reasonably well for estimation of the ch → hex and hex → orto phase transitions at osmotic pressures quantitatively consistent with experiments. A more consistent determination of the phase transition based on no additional assumptions or approximations could proceed from the free-energy landscape computed by enhanced sampling techniques^63, 85–87^.

### Order parameters of water

**Figure 5:**
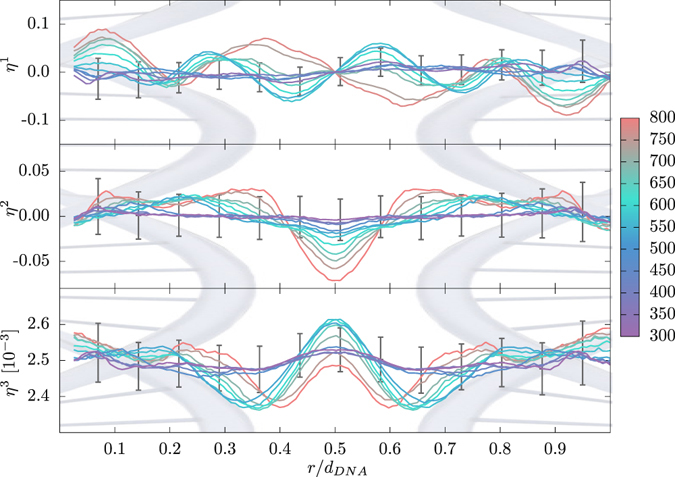
Order parameters ${\eta }^{\mathrm{(1,2,3)}}$ between pairs of DNA molecules. For better comparison, the distances are scaled with DNA interaxial spacings, i.e., values *r*/*d*_*DNA*_ = 0 and 1 correspond to COM of the first and second DNA molecule in the pair, respectively. The results are shown for DNA arrays with hex lattice and Na^+^ counterions at various DNA concentrations indicated by the color palette. The error bars represent the standard deviation.

Local ordering of interstitial water molecules residing between the DNAs is examined by considering the orientational order parameters ${\eta }^{\mathrm{(1},2,\mathrm{3)}}$, defined as ${\eta }^{\mathrm{(1)}}=\langle \cos \,\alpha \rangle$, ${\eta }^{\mathrm{(2)}}=\frac{1}{2}\langle 3\,{\cos }^{2}\,\alpha -1\rangle$, and ${\eta }^{\mathrm{(3)}}=\langle {Q}_{ij}\rangle =\langle {\sum }_{l}\,{q}_{l}{r}_{il}{r}_{jl}\rangle$ (see SI). The first two order parameters measure the average orientation of the dipole and the uniaxial quadrupole moments, being equal to ${\eta }^{\mathrm{(1,2)}}=0$ for the random orientation in the bulk. The third order parameter quantifies the total quadrupole moment and has nonzero value in the bulk. We compute all three order parameters along the axis of a given DNA pair. Thus, *α* denotes the angle between the dipole moment of a water molecule and the shortest vector pointing to the nearest DNA neighbor, while *ij* is the direction along the DNA pair. We consider only water molecules located in a cuboid (defined by DNA-DNA interaxial spacing, DNA diameter and its long axis) between a given DNA pair. Additionally, we scale the distances between the DNA pair so that values *r*/*d*_*DNA*_ = 0 and 1 correspond to COM of the first and the second DNA molecule in the pair, respectively. Figure 5 shows the order parameters ${\eta }^{\mathrm{(1,2,3)}}$ for the hex lattice with Na^+^ counterions after binning the water molecules according to their position along the DNA pair. Obviously on increase of the DNA concentration the local ordering of water molecules, as quantified by the order parameters ${\eta }^{\mathrm{(1,2,3)}}$, shows distinct features and noticeable symmetry. While ${\eta }^{\mathrm{(2,3)}}$ are obviously symmetric w.r.t. the midpoint and indicate progressive orientational layering, ${\eta }^{\mathrm{(1)}}$ is clearly antisymmetric and quantifies the ordering strength of the apposed DNA surfaces. ${\eta }^{\mathrm{(2)}}\ge 0$ for vicinal water indicates that it is oriented preferentially perpendicular to the DNA surface, while ${\eta }^{\mathrm{(2)}}\le 0$ for the interstitial water indicates it exhibits an almost uniform orientation. This tallies favourably also with water ordering and resulting hydration forces between lipid membranes and polar surfaces in general^80, 88^ and we consequently conclude that the hydration forces contribute essentially also to the osmotic pressure at large DNA concentrations. This conclusion is further supported by comparing the total osmotic pressure in the system consisting of DNA and the aqueous solution, with the one obtained for DNA only, without any contribution of the solvent (see SI). Not only is the latter smaller, but can even show an opposite (negative) sign, which indicates that the aqueous solvent adds an essential contribution to the balance of forces in DNA arrays based on the water-mediated hydration interaction, as has been demonstrated in previous experiments on DNA arrays^4, 89^.

**Figure 6:**
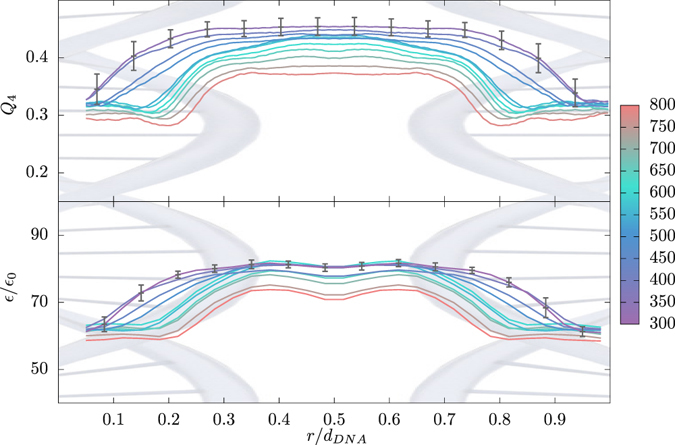
Tetrahedrality *Q*_4_ (top) and relative permittivities (bottom) of water between pairs of neighboring DNA molecules. The results are shown for DNA assemblies in the hex lattice and Na^+^ as counterions, at various DNA concentrations. The symbols are the same as in Fig. 5.

To assess the state of the hydrogen bond network connectivity for water molecules in the DNA subphase, we examine the corresponding *tetrahedrality order parameter*, *Q*_4_, as defined by Debenedetti *et al*.^90^. It quantifies the local geometric tetrahedral order of the first solvation shell of a given molecule, with *Q*_4_ = 1 corresponding to a perfect tetrahedral geometry and *Q*_4_ = 0 to an ideal, disordered gas. For water molecules adjacent to the DNA molecule that would not be able to form a full hydrogen bonded network with other water molecules, it is quite likely that they will share an electronegative DNA atom as a substitute for the missing tetrahedrally coordinated water neighbor, and thus as much as possible reconstitute the original hydrogen-bond network^91^. With this in mind, we calculate *Q*_4_ by actually considering four nearest neighbors irrespective of their identity, *i*.*e*., whether they pertain to water molecules or indeed to DNA atoms. The results (Fig. 6) show that even with this in mind, at very high DNA concentrations the tetrahedral order parameter is appreciably decreased, i.e., the *local structure of water is significantly perturbed*. Similar reasoning applies also to the appropriately defined *local dielectric constant* of water (Fig. 6), which we calculate within the Kirkwood theory where it is related to the average vector sum of the dipole moments of a water molecule centered in a spherical region dug into a solvent continuum (for details see ref. 48). Clearly the decrease of the local dielectric response for water vicinal to DNA indicates the perturbations wrought by the DNA surface that immobilize the water molecules in its vicinity, with the effect extending to a couple of layers away from the molecular surface. This too is consistent with the emergence of the hydration forces at small intersurface separations operating over a range of several molecular layers of water^4, 89^.

## Conclusions

We investigated the couplings between different kinds of interactions and order in the high density DNA mesophases, as well as phase transitions between them, by carrying out a number of multiscale simulations of large systems containing an array of 16 DNA molecules. In particular, the hex and the orto phases were identified and characterized, together with the phase transition between them by invoking the general Lindemann criterion as a proxy for the transition point. Furthermore, we computed the EoS in the high density DNA concentration regime in the presence of mono (Na^+^) as well as multivalent (spermidine) counterions, exhibiting typically a smaller osmotic pressure at the same background salt and DNA conditions, indicating the existence of an attractive interaction (in fact, diminished repulsion), possibly related to the bridging configurations of the multivalent ion between the vicinal DNA surfaces. In addition to the positional and orientational order of DNA molecules, we also studied the related local order parameters of water, its local dielectric profile and the occupancy and the residence times of the ions and water. The former are consistent with a predominant contribution of hydration forces to the DNA EoS at high densities, known from experimental studies of DNA arrays, a conclusion that we substantiate also by considering the EoS of the same system but without the contribution of the aqueous solvent, that typically shows a lower osmotic pressure, sometimes even with the opposite sign. Our conclusion on the role of hydration forces is not incompatible or even less, contrary to the role of electrostatics in the condensation of DNA arrays explored in analytical models^21^ or indeed previous atomistic simulations^42^, since water contributes substantially to mutual repulsion of DNA, while the attraction leading to DNA condensation is of course electrostatic in origin, be it of the correlation^19, 92^ or the bridging type^83, 84^.

In our future work, we intend to determine the point of phase transition more precisely and reliably from the free-energy landscape computed by enhanced sampling techniques^63, 85–87^. Needless to say, this will require much more computational time and computer resources in general.

## Electronic supplementary material

Supplementary Information: Order and interactions in DNA arrays: Multiscale molecular dynamics simulation
