# Supplementary material for: Order and interactions in DNA arrays: Multiscale molecular dynamics simulation
Source: Sci Rep. 2017 Jul 6;7:4775. doi: 10.1038/s41598-017-05109-2 (PMC5500594; doi:10.1038/s41598-017-05109-2)
Supplement: Supplementary file 1 — Supplementary Information: Order and interactions in DNA arrays: Multiscale molecular dynamics simulation [file 41598_2017_5109_MOESM1_ESM.pdf]

# Supplementary Information: Order and interactions in DNA arrays: Multiscale molecular dynamics simulation

Julija Zavadlav,[1,2,a] Rudolf Podgornik,[2,3,b] and Matej Praprotnik[1,2,c]

[1] *Department of Molecular Modeling, National Institute of Chemistry, Hajdrihova 19, SI-1001 Ljubljana, Slovenia*

[2] *Department of Physics, Faculty of Mathematics and Physics, University of Ljubljana, Jadranska 19, SI-1000 Ljubljana, Slovenia*

[3] *Theoretical Physics Department, J. Stefan Institute, Jamova c. 39, SI-1000 Ljubljana, Slovenia*

[a] Current address: Chair of Computational Science, ETH Zurich, Clausiusstrasse 33, CH-8092 Zurich, Switzerland

[b] Electronic mail: rudolf.podgornik@fmf.uni-lj.si

[c] Electronic mail: praprot@cmm.ki.si

## I. Interaction phenomenology

Monovalent salts, apart from their specific interaction with the DNA surface[1,2], provide usually a screening electrolyte medium for *electrostatic repulsion* between highly charged dsDNAs, while trivalent cations such as $\mathrm{CoHex}^{3+}$ and/or $\mathrm{Spd}^{3+}$ at sufficiently large concentrations can lead to dsDNA condensation by turning electrostatic repulsion into a *counterion mediated attraction*, despite DNA's high negative charge[3–7], enforcing a phase separation from the bathing electrolyte. While high valency is a necessary condition for attractive interaction, it is not exhaustive as e.g. CoHex is more efficient as a condensing agent then spermidine, though they are both equally charged[1]. On the other hand, monovalent cations such as $\mathrm{K}^{+}$ can in their turn act as condensing agents for quadruple stranded DNA[8]; furthermore, whereas some divalent ions such as $\mathrm{Cd}^{2+}$ and $\mathrm{Mn}^{2+}$ can under certain circumstances condense the double stranded DNA, other divalent salts, containing e.g. $\mathrm{Mg}^{2+}$ cation, again act plainly as a screening electrolyte medium for dsDNA, but at the same time invariably condensing the triple stranded DNA[9]. These features make the unique identification of the *ultimate mechanism* of counterion-mediated DNA condensation difficult to pin down.

The phenomenology of DNA-DNA interactions, as revealed by the EoS experiments, is complicated and defies a single classification scheme, depending on a multiplicity of conditions. While it is clear that purely mean-field electrostatic theories based on the Poisson-Boltzmann phenomenology cannot predict anything but repulsion[5], venturing beyond this framework can be pursued on different levels. By concentrating on the non-mean-field correlation effects, as first suggested by Rouzina and Bloomfield[10], the *Wigner crystal* approach[11] and the *strong coupling* approach[12] as well as their varieties, such as the charge density wave[13] and ion bridging model[14,15], all single out the universal features of higher valency counterions, based on their valency alone, as responsible for the attractions that they mediate. On the other hand, the *tight counterion binding* approach[1] was the first to introduce non-electrostatic interactions as governing the distribution of the counterions on the surface of DNA, while preserving the electrostatics as the mechanism mediating the interactions between thus decorated DNAs. The furthest deviation from the predominant role of electrostatics was the proposition by Rau and Parsegian[16] that water-mediated interactions present the most important source of DNA attractions and that the way to understand them is to abolish the implicit dielectric continuum model for the solvent.

## II. Additional simulation details

DNA molecules are always described on the atomistic level and modelled with the AMBER 03 force field[17]. On the atomistic scale, the water molecules are modelled with a TIP3P model[18]. For the $\mathrm{Na}^{+}$ and $\mathrm{Cl}^{-}$ ions we first test the standard AMBER 03 parameters, but find that the DNA molecules substantially deviate from their reference lattice positions, in accordance with Ref.[19]. There, DNA molecules under similar conditions were observed to form clusters, contrary

to the experimental observations. Furthermore, discrepancies were also found for the osmotic pressure of the DNA arrays. Thus, we follow the guidance of Ref.[19] and instead use the Joung and Cheatham parameters[20] with additional corrections to $Na^{+}$-phosphate interaction. The $Spd^{3+}$ molecules are modeled only on the atomistic level (not leaving the atomistic domain) with the parameters obtained with AmberTools program[21], employing a standard procedure for obtaining custom molecule force field parameters. The water molecules and ions ($Na^{+}$ and $Cl^{-}$) can change their resolution adaptively on the fly between the atomistic and coarse-grained regions. The effective interactions on the coarse-grained scale are determined via Iterative Boltzmann Inversion method[22] that is incorporated into the STOCK web toolkit[23].

For each lattice type and initial DNA-DNA separation the DNA molecule is translated and rotated to form an array. In the hexagonal configuration, the DNA molecules are rotated by $2\pi/3$ with respect to each other. In the orthorhombic configuration, the four nearest neighbors are rotated by $\pi/2$, whereas the two further away are at the non-optimal orientation ($\triangle\varphi = 0$). The array is solvated with the $1$ M salt solution with added counterions for charge neutrality. For the evaluation of the sufficient number of DNA molecules in the array we perform additional simulations where DNA arrays are composed of $9, 25,$ and $36$ DNA molecules.

The simulations are performed in a rhombic prism (with $60^{\circ}$ inclination angle) simulation box with periodic boundary conditions in all directions and with a minimum image convention. The simulation box size in the x,y-directions varies with lattice spacing $a$. In the z-direction, it is fixed and corresponds to exactly one DNA pitch, i.e., 3.4 nm. The periodic boundary condition is applied also to the DNA helix. In particular, we use patches, i.e., corresponding intramolecular DNA interactions defined by bond, angle, and dihedral interaction potentials, to connect each strand to its periodic image along the z-axis. While it is more customary to use the experimentally determined DNA structures for the initial structure, we opt here for a generic one. In particular, the initial atomic coordinates of a B-form DNA with $10$ base-pair sequence (5'-CTCTCGCGCG-3') molecule are generated using the 3D-DART Web server[24]. With this choice we avoid the possible strains that could occur at the periodic links of both chains. The atomistic region is a rhombic prism and for the hexagonal latices coincides with the semipermeable membrane. For the orthorhombic lattices the size is set to encompass the whole membrane, which is tilted at a different angle (see Figure S1). The width of hybrid and coarse-grained regions are set to $0.9$ nm for all cases. The resolution region boundaries are static during the simulation run. The thermodynamic forces applied in the adaptive resolution scheme (AdResS) are depicted in Figure S2 for water, $Na^{+}$ and $Cl^{-}$. They are slightly modified form Ref.[25] due to different model parameters.

## III. Osmotic pressure calculation

The osmotic stress methodology to determine the osmotic pressure in the DNA subphase relies on the equivalence between the mechanical and the osmotic forces acting on a semipermeable

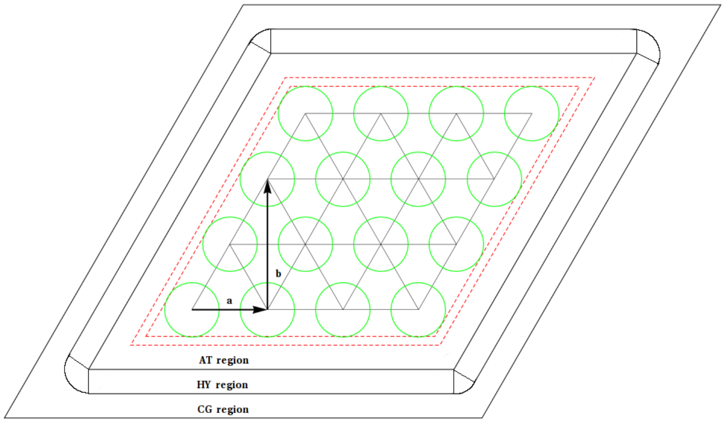


FIG. S1. Schematic representation of the system setup for the hexagonal and ortorhombic phases (top and bottom, respectively). The lattice parameter $a$ ranges from 2.0 to 3.6 nm, whereas the lattice parameter $b$ is set so that the ratio $b/a$ is equal to $\sqrt{3}$ and 1.43 in the hexagonal and ortorhombic phases, respectively. The red dotted lines denote the region, where the wall force acts.

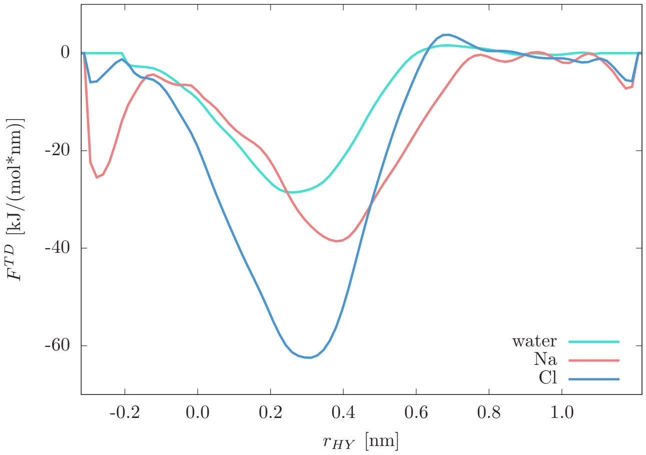


FIG. S2. Thermodynamic forces applied to molecules in the hybrid region as a function of distance $r_{HY}$ from atomistic region. The $r_{HY} = 0$ and $r_{HY} = 0.9$ nm correspond to the atomistic-hybrid and the hybrid-coarse-grained boundaries, respectively.

membrane, the latter controlled by a water soluble polymer (usually polyethyleneglycol - PEG)[26], that exerts a uniform compression on the DNA subphase[16].

In MD simulations, the osmotic pressure between pure water and salt solution region can be calculated by introducing a semi-permeable membrane[27,28]. We calculate the osmotic pressure of a DNA array in a similar way by placing a semi-permeable membrane around the set of DNA molecules. The membrane is modelled by a repulsive part of the 10-4 Lennard-Jones interaction

of the following form

$$U_{wall}(x) = 2\pi\rho\epsilon\left(\frac{2\sigma^{12}}{5x^{10}} - \frac{\sigma^6}{x^4}\right), \tag{S1}$$

where $x$ is a perpendicular distance to the wall, $\sigma$ and $\epsilon$ are the 12-6 Lenard-Jones parameters and $\rho$ is the wall density. The interaction is applied to the DNA backbone atoms and thus prevents a given DNA molecule from crossing the wall, whereas the solvent molecules (water, $Na^+$, and $Cl^-$ ions) can freely pass through. The system inside walls is thus open, i.e., semi grand canonical, even though the total system is closed. The osmotic pressure of the DNA array is then calculated as $\overline{F_{wall}}/A$, where $\overline{F_{wall}}$ is the mean force on the wall and $A$ is its area.

In DNA arrays of hexagonal symmetry, the osmotic pressure can be equivalently plotted as a function of the interaxial spacings between nearest neighbors or the DNA density, while in the orthorhombic phase the DNA-DNA spacings are not all equal and thus the osmotic pressure-density plot offers a better comparison between the two lattices. Note that the DNA density is defined as an average inside the semi-permeable membrane and depends on its size. Additionally, the DNA-DNA separation varies during the simulation and deviates (especially for low densities) from the initially set value.

## IV. Additional Results

### Size variation

The experimentally observed DNA liquid crystals contain a large number of DNA molecules, while in the simulations we can only simulate a few. Thus, it is important to first evaluate the size effects on the osmotic pressure. We tested four sizes of DNA assemblies that contain $9$, $16$, $25$, and $36$ DNA molecules. In Figure S3, we plot the osmotic pressure for all three cases as a function of time. The simulations are performed under the same conditions (hexagonal lattice, $a = 2.1$ nm, $Na^+$ counterions). The average osmotic pressure is approximately the same for all three sizes, while the fluctuations are larger for smaller systems. Note that the pressure is increasing in the first $2$ ns. For this reason, we average the rest of the results only over the last $20$ ns, i.e. the equilibration runs are $2$ ns long while the production runs are $20$ ns.

### Determination of phase transition via Lindemann criterion

The translational and orientational orders of the DNA assemblies are also quantified by the normalized root-mean-square deviation $\sigma_{\mathbf{r}}/a$ and the rotational root mean square deviations $\sigma_\varphi/\pi$ as a function of pressure, respectively (see the main article). The results are depicted in Figure S4.

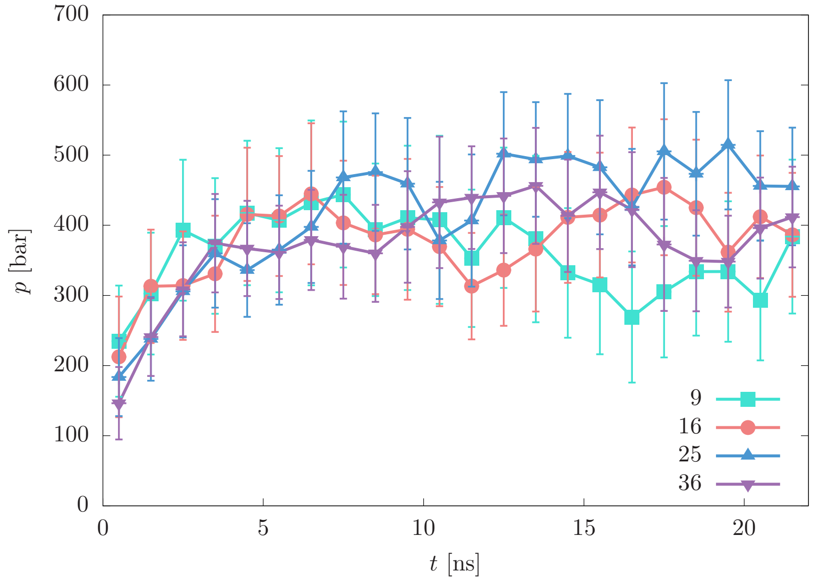


FIG. S3. Time dependence of the osmotic pressure on the DNA assembly for different system sizes, i.e., the assembly of 9, 16, and 25 DNA molecules. In all cases, the system is net neutralized with $Na^+$ ions, while the DNA molecules are arranged into the hexagonal lattice with a lattice spacing of 2.1 nm.

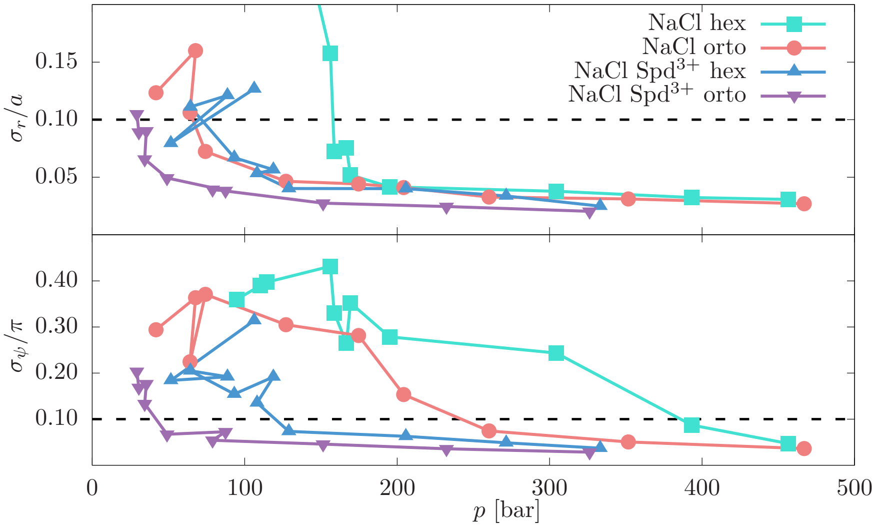


FIG. S4. Positional (divided by lattice parameter $a$) and rotational (divided by $\pi$) standard deviations of mean values as a function of pressure for the hexagonal and orthorhombic lattice with pure NaCl and mixed NaCl and $Spd^{3+}$ counterions. The horizontal dotted lines indicate the typical 0.1 *Lindemann threshold value.*

## Local pressure / Hydration force

The $p_{yy}$ component of the local pressure tensor within the membrane walls is calculated along the $y$-axis (see Figure S6) using the Method of Planes (MOP) definition[29,30]. The profiles of the pressure are calculated by splitting the axis into slabs. The slab component is composed of a kinetic contribution $p_{yy}^{kin}(y)$ and a virial part $p_{yy}^{pot}(y)$ defined as

$$p_{yy}^{kin}(y) = \frac{1}{A}\sum_i \left\langle \frac{p_{y,i}p_{y,i}}{m_i} sgn(y_i - y) \right\rangle \tag{S2}$$

and

$$p_{yy}^{pot}(y) = \frac{1}{2A}\sum_i \left\langle f_{y,i} sgn(y_i - y) \right\rangle . \tag{S3}$$

The $\langle \ldots \rangle$ denotes the configurational average and the $m_i$, $p_i$ and $f_i$ are the mass, velocity and total force on particle $i$, respectively. Figure S5 shows the difference in pressure contributions of the total system $p_{yy}^{all}$ and DNA atoms alone $p_{yy}^{DNA}$. The pressure profiles have an oscillating shape with minima corresponding to positions of the DNA molecules. The average $\bar{p}_{yy}^{DNA}$ component of

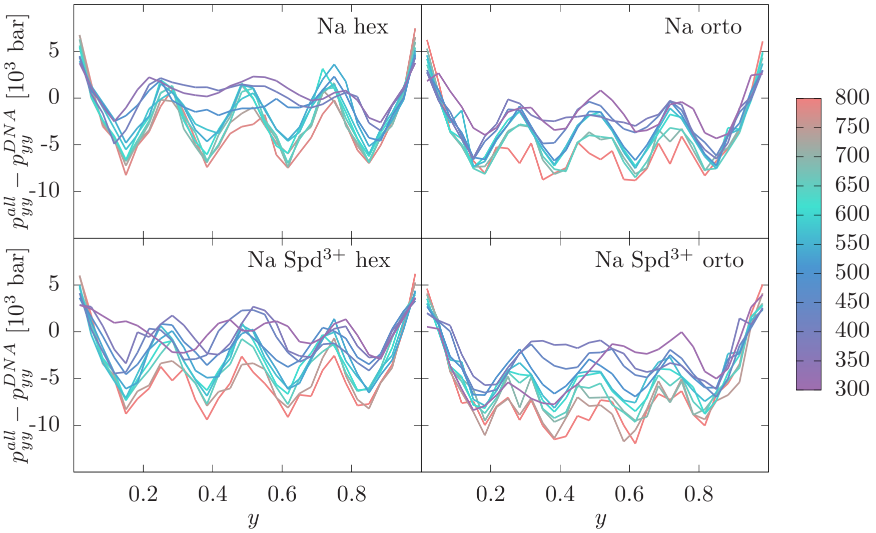


FIG. S5. Pressure difference $p_{yy}^{all} - p_{yy}^{DNA}$ of the total system, DNA plus aqueous solution, and DNA alone. For better comparison the distances are scaled with membrane thickness in $y$ direction $d_y^{wall}$, i.e., values $y = 0$ and 1 correspond to bottom and top membrane wall, respectively. The results are shown at various DNA concentrations indicated by the color palette.

the local pressure tensor within the membrane walls is computed by averaging over $y$. Figure S7 shows $\bar{p}_{yy}^{DNA}$ of DNA atoms as a function of DNA density, compared to the total osmotic pressure shown in Figure 2, for the case of a single type of salt. As the pure DNA contribution to the

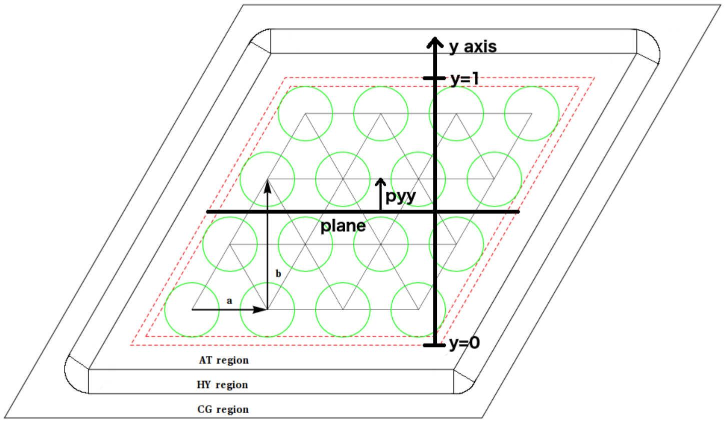


FIG. S6. Schematic representation of the calculation of the pressure $p_{yy}(y)$ showing the $y$-axis, the orthogonal plane and the direction of the pressure tensor component.

osmotic pressure cannot be computed via the average force exerted on the membrane because the DNA structure is not stable without the presence of water, the DNA contribution to the osmotic pressure is computed via the MOP instead. Obviously, there is a substantial difference between

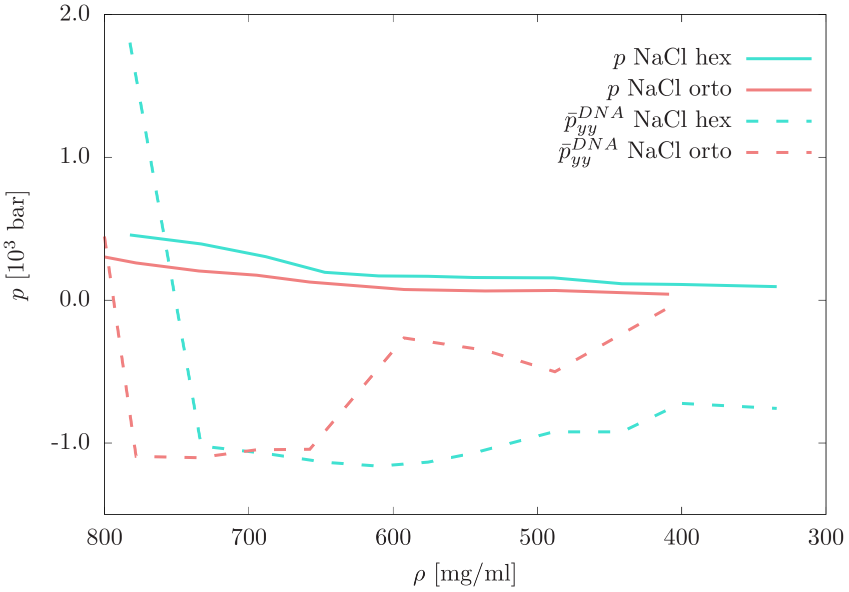


FIG. S7. The total osmotic pressure $p$ and the average $\bar{p}_{yy}^{DNA}$ component of the local pressure tensor of DNA atoms, as a function of DNA density in the case of hexagonal and ortorhombic lattice for the NaCl case. The pure DNA osmotic pressure in the case without the solvent contribution is smaller then the total osmotic pressure.

the total osmotic pressure and the pure DNA contribution, that can even become negative, in complete analogy with the case of interacting hydrated phospholipid membranes [31]. This difference

indicates that water contributes fundamentally to the force equilibrium in the DNA array and the identification of the hydration force as its major component is thus vindicated.

## Order parameters

The water order parameters are shown in Figures S8-S10. In the order parameter $\eta^{(3)} = \langle Q_{ij} \rangle = \langle \sum_l q_l r_{il} r_{jl} \rangle$, the index $l$ runs over the water hydrogens with charge $q_l$ and $r$ is the distance between the hydrogen and oxygen atom of the considered water molecule. $\langle \ldots \rangle$ denotes the average over trajectory and over all the DNA pairs.

Water in the DNA subphase tends to limit to its bulk properties for concentrations below $\sim$ 300 mg ml$^{-1}$, and deviates significantly from the full bulk properties at concentrations above $\sim$ 600 mg ml$^{-1}$.

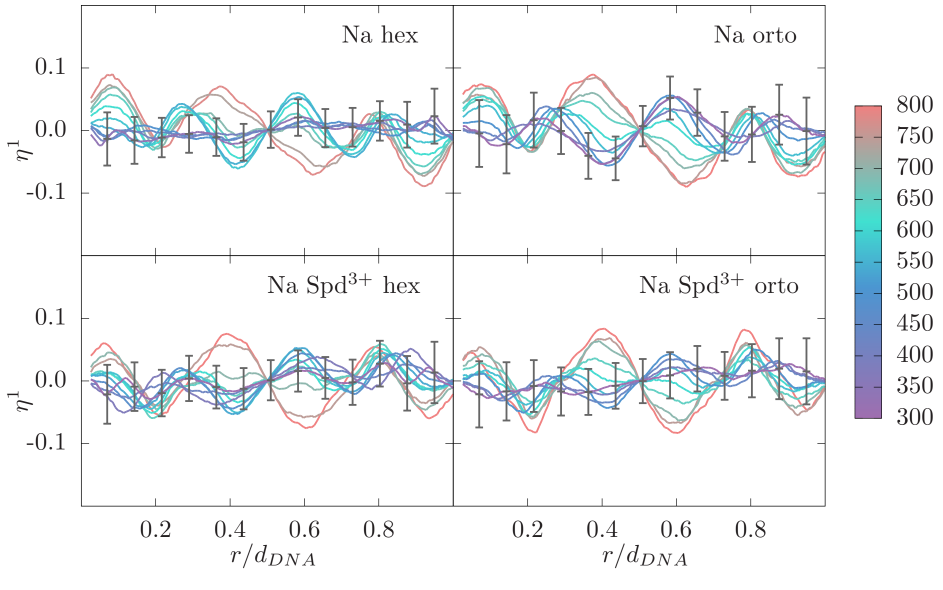


FIG. S8. Water order parameters $\eta^{(1)}$ between pairs of DNA molecules. For better comparison the distances are scaled with DNA separations, i.e., values $r/d_{DNA} = 0$ and 1 correspond to COM of the first and second DNA molecule, respectively. The results are shown at various DNA concentrations indicated by the color palette. The error bars represent the standard deviation of the measurements.

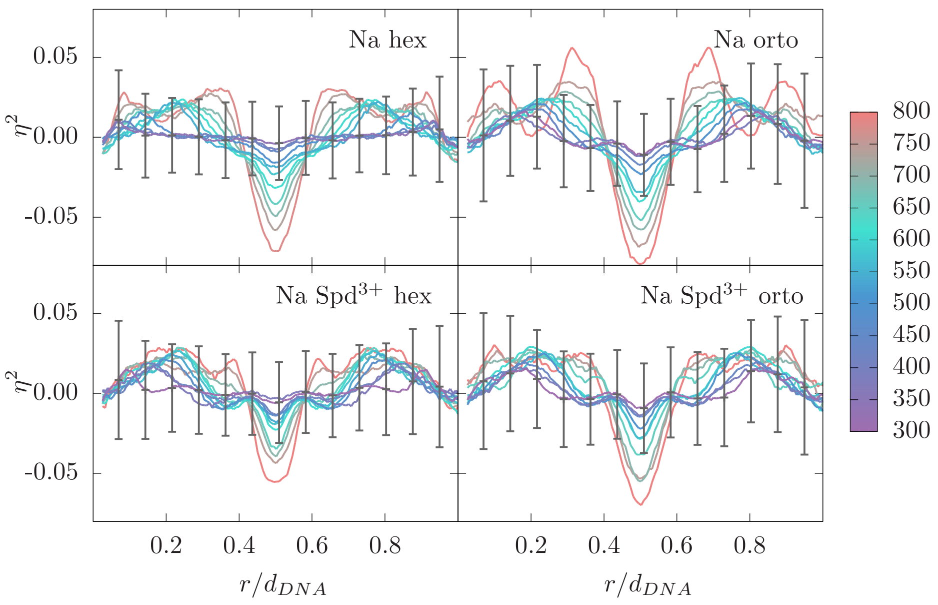


FIG. S9. Water order parameters $\eta^{(2)}$ between pairs of DNA molecules. The representation is the same as in Figure S8.

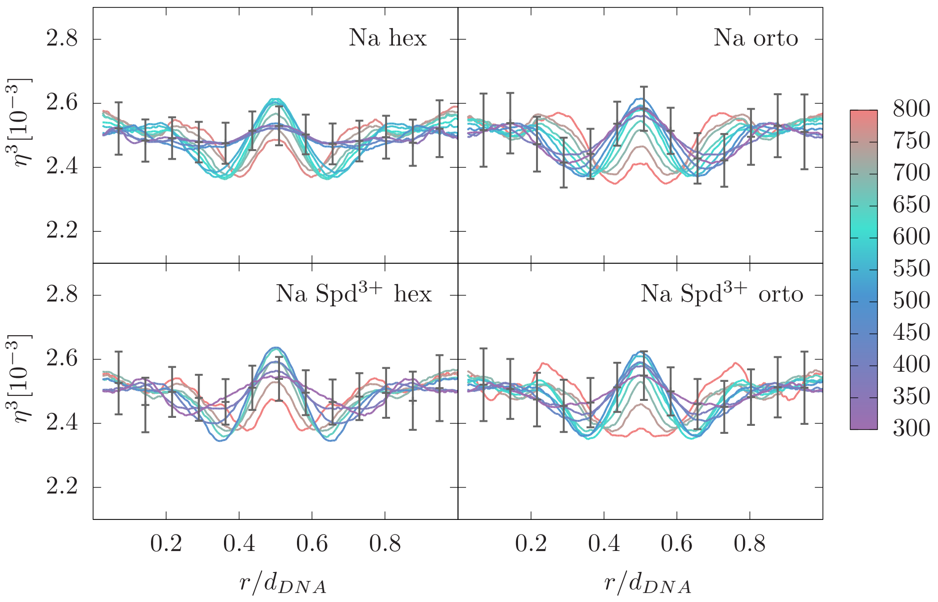


FIG. S10. Water order parameters $\eta^{(3)}$ between pairs of DNA molecules. The representation is the same as in Figure S8.

## Tetrahedral order

The tetrahedrality order parameter $Q_4$ is shown in Figure S11 and is defined as[32]

$$Q_4 = 1 - \frac{3}{8}\sum_{i=1}^{3}\sum_{j=i+1}^{4}\left(\cos\theta_{ijk} + 1/3\right)^2 , \tag{S4}$$

where $i$ is the oxygen atom of the reference water molecule and $\theta_{ijk}$ the angle between vectors $\mathbf{r}_{ij}$ and $\mathbf{r}_{ik}$, where $j$ and $k$ are the oxygen atoms of nearest neighbors of central water $i$. The sum runs over distinct pairs of the four closest neighbors, i.e., over six oxygen-oxygen-oxygen angles. The spatially varying $Q_4$ is computed by discretizing distances from the DNA molecule into bins and averaging over water molecules that fall into a corresponding bin.

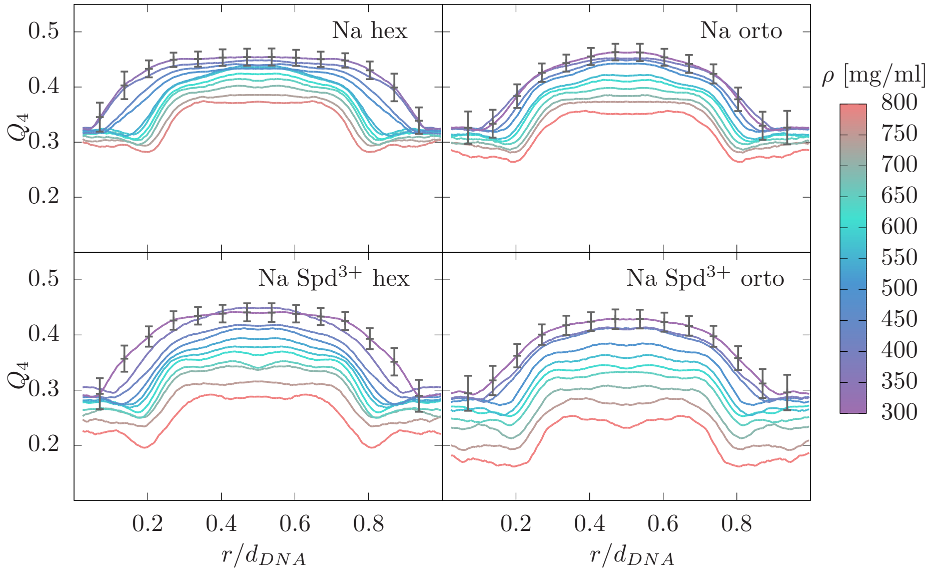


FIG. S11. Tetrahedrality order parameter $Q_4$. The representation is the same as in Figure S8.

## Relative permittivity of water

Figure S12 shows the relative permittivities of water as defined in Ref.[25].

## Occupancy and residential times of solvent entities

We compute the average occupancy and residence times of counterions $Na^+$ and $Spd^{3+}$ and oxygen atoms of water as detailed in Ref.[25]. The calculated values are shown in Figures S13-S16.

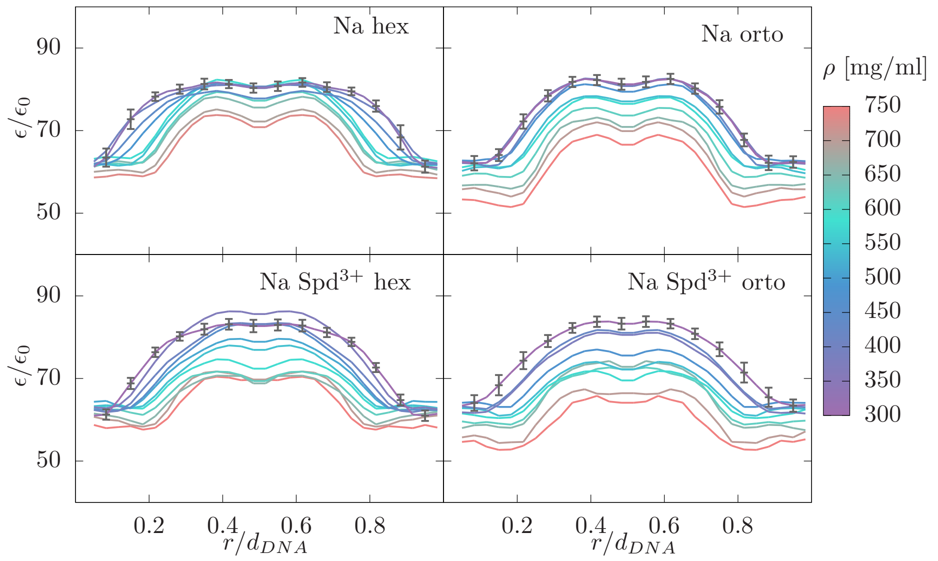


FIG. S12. Relative permittivities of water. The representation is the same as in Figure S8.

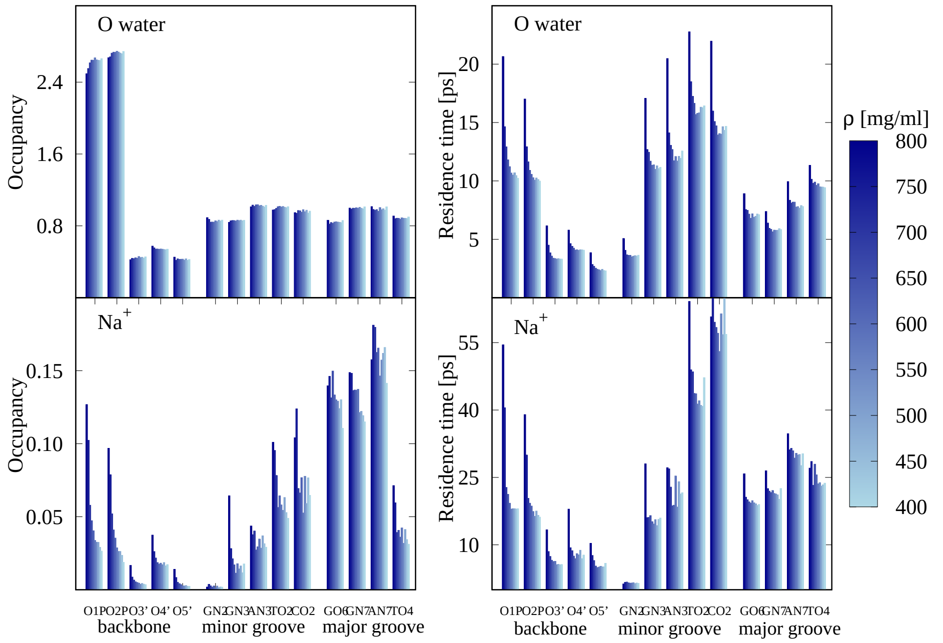


FIG. S13. Average occupancy (left) and residence time (right) of oxygen atoms of water (top) and $Na^+$ ions (bottom) for the hexagonal lattices with only $Na^+$ counterions. The calculation is performed only on the atoms in the first solvation shell of the electronegative atoms of DNA. Results are shown for various DNA concentrations marked with a different shade as shown on the color palette.

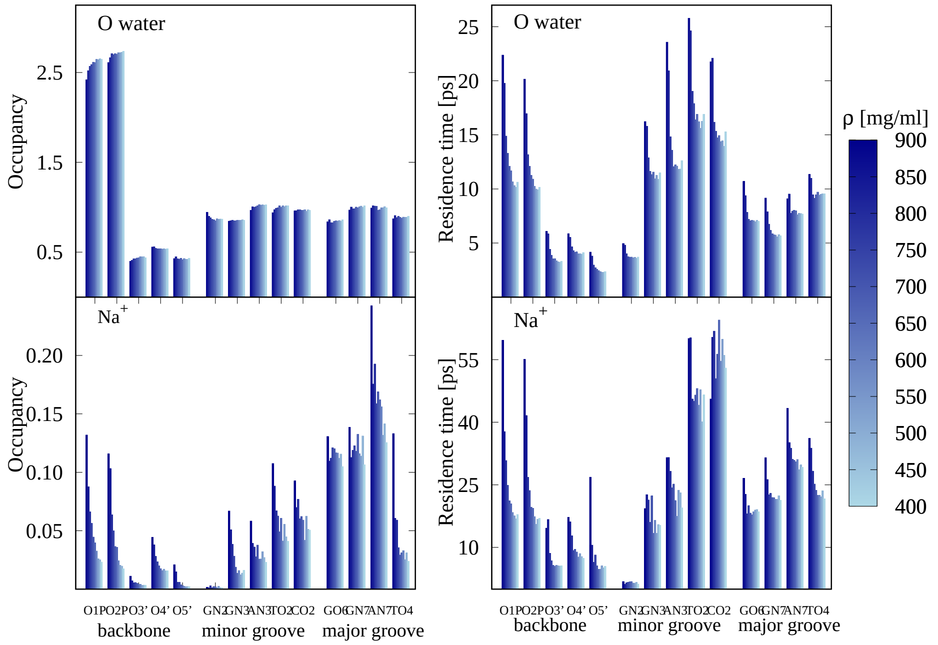


FIG. S14. Same as Figure S13 for the orthorhombic lattices.

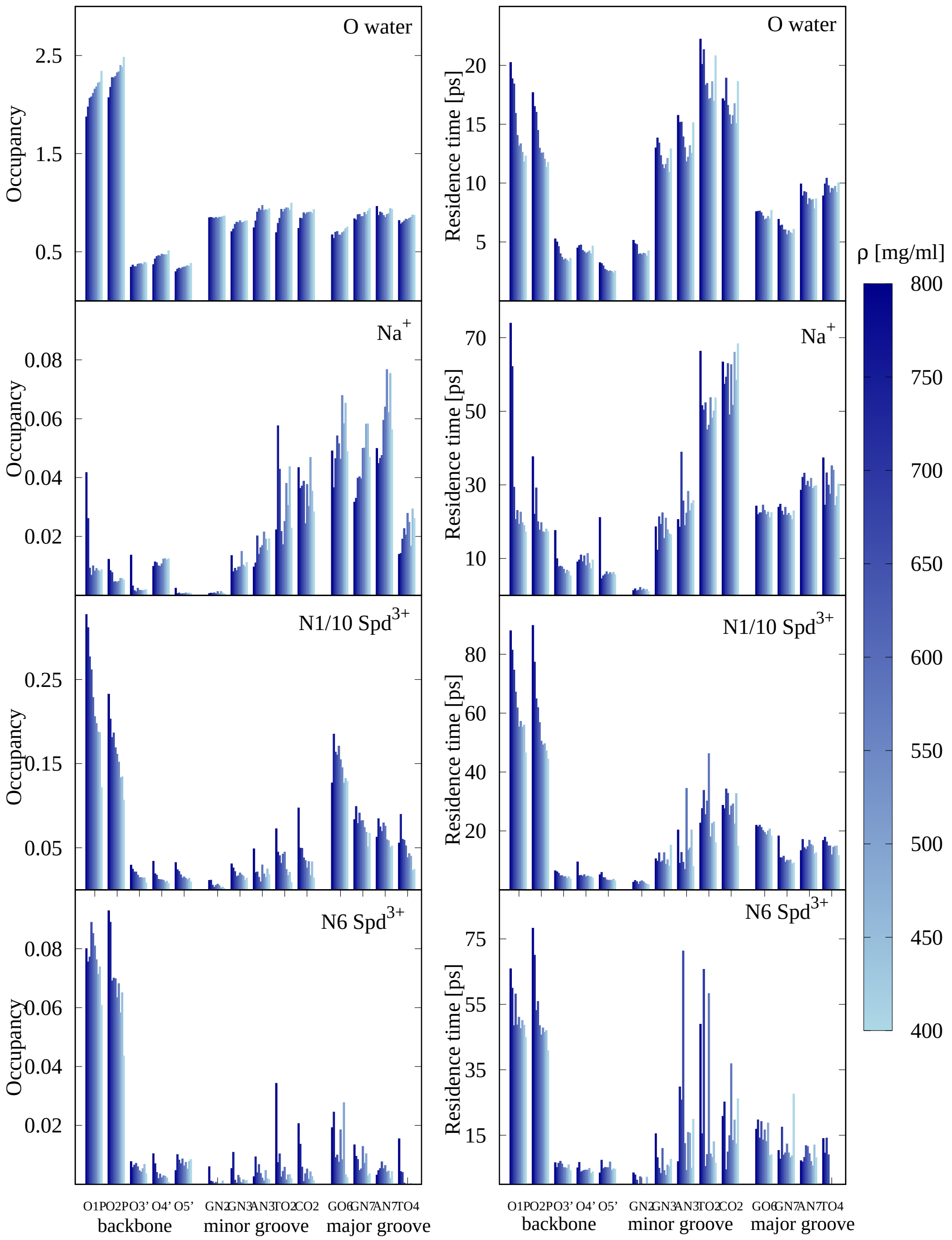


FIG. S15. Same as Figure S13 for the hexagonal lattices with $Na^{+}$ and $Spd^{3+}$ counterions.

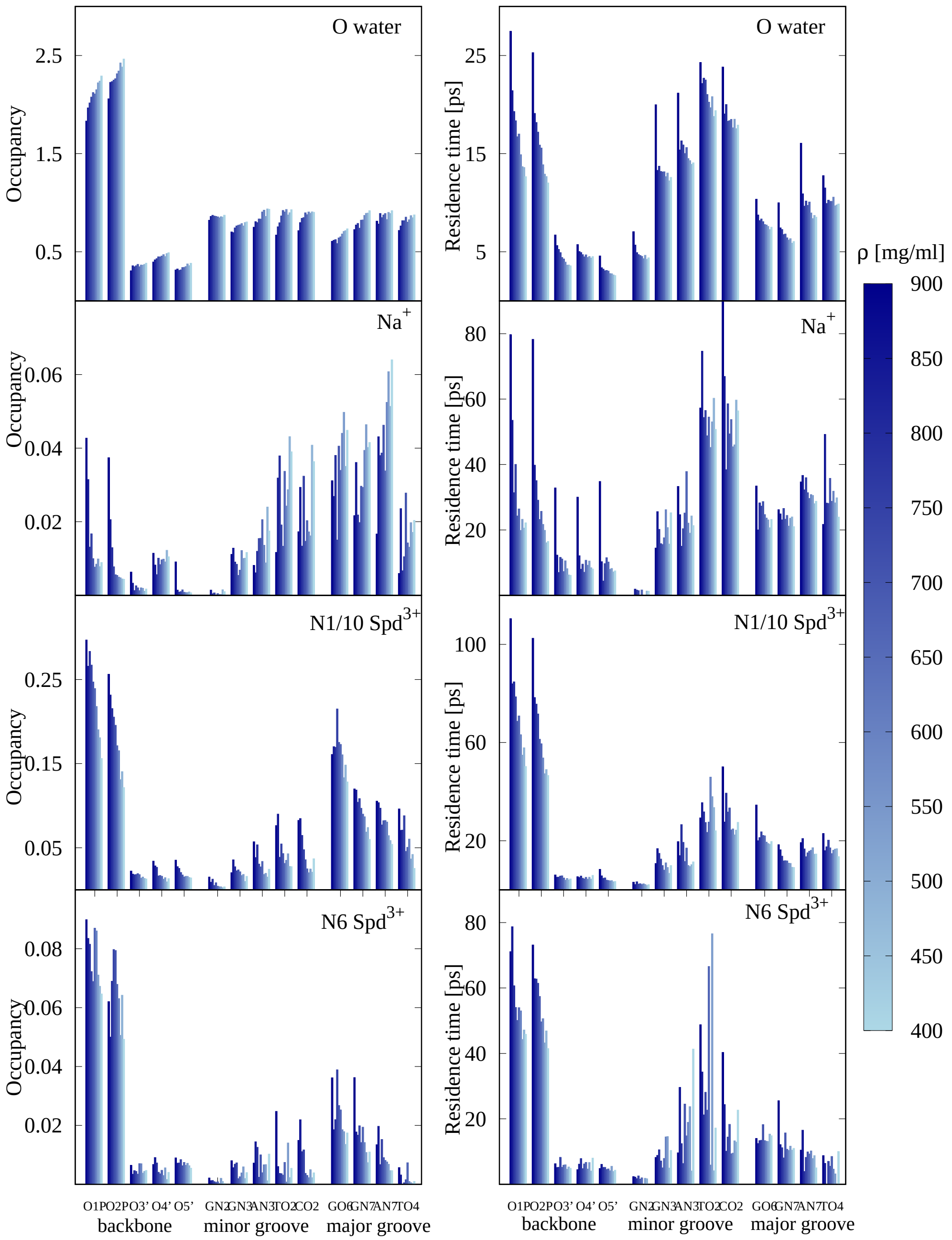


FIG. S16. Same as Figure S13 for the orthorhombic lattices with $Na^{+}$ and $Spd^{3+}$ counterions.

## References


[1]Kornyshev, A. A., Lee, D. J., Leikin, S. & Wynveen, A. Structure and Interactions of Biological Helices. *Rev. Mod. Phys.* **79**, 943–996 (2007).

[2]Podgornik, R., Rau, D. & Parsegian, V. Parametrization of direct and soft-undulatory forces between DNA double helical polyelectrolytes in solutions of several different anions and cations. *Biophys.J.* **66**, 962 – 971 (1994).

[3]Bloomfield, V. DNA condensation. *Curr. Opin. Struct. Biol.* **6**, 334–341 (1996).

[4]Bloomfield, V. DNA condensation by multivalent cations. *Biopolymers* **44**, 269–282 (1997).

[5]Gelbart, W. M., Bruinsma, R. F., Pincus, P. A. & Parsegian, V. A. DNA-inspired electrostatics. *Physics Today* **53**, 38–44 (2000).

[6]Qiu, X. *et al.* Salt-dependent DNA-DNA spacings in intact bacteriophage $\lambda$ eflect relative importance of DNA self-repulsion and bending energies. *Phys. Rev. Lett.* **106**, 028102 (2011).

[7]Kornyshev, A. A. Physics of DNA: unravelling hidden abilities encoded in the structure of the most important molecule. *Phys. Chem. Chem. Phys.* **12**, 12352–12378 (2010).

[8]Yasar, S. *et al.* X-ray characterization of mesophases of human telomeric G-quadruplexes and other DNA analogues. *Sci. Rep.* **6**, 27079 (2016).

[9]Qiu, X., Rau, D. C. & Parsegian, V. Divalent counterion-induced condensation of triple-strand DNA. *Proc. Natl. Acad. Sci. USA* **107**, 2148221486 (2010).

[10]Rouzina, I. & Bloomfield, V. A. Macroion Attraction Due to Electrostatic Correlation between Screening Counterions. 1. Mobile Surface-Adsorbed Ions and Diffuse Ion Cloud. *J. Phys. Chem.* **100**, 9977–9989 (1996).

[11]Grosberg, A., Nguyen, T. & Shklovskii, B. Colloquium: the physics of charge inversion in chemical and biological systems. *Rev. Mod. Phys.* **74**, 329–345 (2002).

[12]Naji, A., Kanduč, M., Forsman, J. & Podgornik, R. Perspective: Coulomb fluids – weak coupling, strong coupling, in between and beyond. *J. Chem. Phys.* **139**, 150901 (2013).

[13]Angelini, T. *et al.* Counterions between charged polymers exhibit liquid-like organization and dynamics. *Proc. Natl. Acad. Sci.* **103**, 79627967 (2006).

[14]Raspaud, A., Olvera de la Cruz, M., Sikorav, J.-L. & Livolant, F. Precipitation of DNA by Polyamines: A Polyelectrolyte Behavior. *Biophys. J.* **74**, 381–393 (1998).

[15]Kanduč, M., Dobnikar, J. & Podgornik, R. Counterion-mediated electrostatic interactions between helical molecules. *Soft Matter* **5**, 868877 (2009).

[16]Rau, D. C. & Parsegian, V. A. Direct measurment of temperature-dependent solvent forces between DNA double helices. *Biophys. J.* **61**, 260–271 (1992).

[17]Duan, Y. *et al.* A point-charge force field for molecular mechanics simulations of proteins based on condensed-phase quantum mechanical calculations. *J. Comput. Chem.* **24**, 1999–2012 (2003).

[18]Jorgensen, W. L., Chandrasekhar, J., Madura, J. D., Impey, R. W. & Klein, M. L. Comparison of simple potential functions for simulating liquid water. *J. Chem. Phys.* **79**, 926–935 (1983).

[19]Yoo, J. & Aksimentiev, A. Improved parametrization of Li+, Na+, K+ and Mg2+ ions for all-atom molecular dynamics simulations of nucleic acid systems. *J. Phys. Chem. Lett.* **3**, 45–50 (2011).

[20]Joung, I. S. & Cheatham, T. E. r. Determination of alkali and halide monovalent ion parameters for use in explicitly solvated biomolecular simulations. *J. Phys. Chem. B* **112**, 9020–9041 (2008).

[21]Case, D. A. *et al.* The Amber biomolecular simulation programs. *J. Comput. Chem.* **26**, 1668–1688 (2005).

[22]Reith, D., Pütz, M. & Müller-Plathe, F. Deriving effective mesoscale potentials from atomistic simulations. *J. Comput. Chem.* **24**, 1624–1636 (2003).

[23]Bevc, S., Junghans, C. & Praprotnik, M. Stock: Structure mapper and online coarse-graining kit for molecular simulations. *J. Comput. Chem.* **36**, 467–477 (2015).

[24]van Dijk, M. & Bonvin, A. M. J. J. 3D-DART: A DNA structure modelling server. *Nucleic Acids Res.* **37**, 235–239 (2009).

[25]Zavadlav, J., Podgornik, R. & Praprotnik, M. Adaptive resolution simulation of a DNA molecule in salt solution. *J. Chem. Theory Comput.* **11**, 5035–5044 (2015).

[26]Cohen, J. A., Podgornik, R., Hansen, P. L. & Parsegian, V. A. A Phenomenological One-Parameter Equation of State for Osmotic Pressures of PEG and Other Neutral Flexible Polymers in Good Solvents. *J. Phys. Chem. B* **113**, 3709?3714 (2009).

[27]Shen, J. W., Li, C., van der Vegt, N. F. A. & Peter, C. Transferability of coarse grained potentials: Implicit solvent models for hydrated ions. *J. Chem. Theory Comput.* **7**, 1916–1927 (2011).

[28]Luo, Y. & Roux, B. Simulation of osmotic pressure in concentrated aqueous salt solutions. *J. Phys. Chem. Lett.* **1**, 183–189 (2010).

[29]Todd, B. D., Evans, D. J. & Daivis, P. J. Pressure tensor for inhomogeneous fluids. *Phys. Rev. E* **52**, 1627–1638 (1995).

[30]Podgornik, R., Åkesson, T. & Jönsson, B. Colloidal interactions mediated via polyelectrolytes. *J. Chem. Phys.* **102**, 9423–9434 (1995).

[31]Schneck, E., Kanduč, M. & Netz, R. R. Hydration repulsion between biomembranes results from an interplay of dehydration and depolarization. *Proc. Natl. Acad. Sci.*. **109**, 1440514409 (2012).

[32]Errington, J. R. & Debenedetti, P. G. Relationship between structural order and the anomalies of liquid water. *Nature* **409**, 318–321 (2001).
